# The prevalence of neck pain and its association with studying device usage and posture among students at the University of Jordan: A cross-sectional study

**DOI:** 10.1371/journal.pone.0326478

**Published:** 2026-05-22

**Authors:** Mohammad Ahmad Al-Shalalfeh, Yazan Zayat, Malak Abu Al Haj, Omar Nached, Mohammad Suleiman, Ahmad Alaudat, Ahmed Salman

**Affiliations:** 1 School of Medicine, The University of Jordan, Amman, Jordan; 2 School of Medicine, The Hashemite University, Zarqa, Jordan; 3 Department of Anatomy and Histology, Faculty of Medicine, The University of Jordan,‌‌ Amman, Jordan; 4 Department of Anatomy, Faculty of Medicine, Menoufia University, Menoufia,‌‌ Egypt; Universidad Santiago de Cali, COLOMBIA

## Abstract

**Background:**

Neck pain is one of the leading causes of discomfort and disability, especially among students who use digital devices and books for studying. This study investigates the prevalence of neck pain among students of the University of Jordan and the association of potential risk factors, including posture and study devices.

**Methods:**

This cross-sectional survey was conducted at the University of Jordan. Data were collected through structured, face-to-face interviews from eligible students stratified across the University’s 20 faculties to ensure proportional representation. Known musculoskeletal disease, congenital anomalies, or prior neck/shoulder trauma or surgery cases were excluded. The questionnaire covered demographics, study behaviors, primary and secondary study utilities, posture, study duration, physical activity, and pain intensity reported based on NRS-11 scoring. Analysis was performed using JASP. We used univariate and multivariate logistic regression.

**Results:**

The data analyzed showed that out of the 507 students, (52.4%) complained of neck pain within the last week, with the most complained site of pain being the neck (56%), and the least being the left shoulder (5.2%). Sitting slouched was associated with approximately two-fold higher odds of reporting neck pain compared to sitting upright with full back support (OR = 2.18, CI [1.18, 4.04], p = 0.013). Using laptops (OR = 2.65, CI [1.45, 4.87], p = 0.002) or tablets (OR = 2.68, CI [1.34, 5.36], p = 0.005) for studying was associated with a nearly 2.7-fold increase in the odds of neck pain compared with using phones. In contrast, studying with books was not significantly associated with neck pain.

**Conclusion:**

Slouched sitting and use of laptops or tablets were associated with higher odds of reporting neck pain, while studying primarily on phones was associated with lower odds of reporting neck pain compared with laptops and tablets. This study identified the urgent need for ergonomic education and interventions to promote healthier study habits and reduce musculoskeletal strain in students.

## Introduction

Neck pain is a major musculoskeletal disorder and a growing concern in modern society that contributes to disability, economic burden, and reduced quality of life [1].

A global study published in 2021 found that neck pain remains one of the leading causes of years lived with disability (YLDs) worldwide. The study reported that in 2020, neck pain affected 203 million people, with age-standardized prevalence rates being higher in females in comparison to males. By 2050, the number of global neck pain cases is projected to increase by 32.5%, accounting for approximately 269 million cases, due to population growth being the primary contributing factor, followed by population aging. Other analyses have confirmed its ranking as the fourth leading cause of disability globally, with lifetime prevalence exceeding 30% [1–3].

Neck pain, typically associated with occupational settings, especially among those who engage in prolonged desk work, is now an emerging concern among university students. The shift toward digital learning, combined with increased sedentary study habits, is associated with a higher incidence of neck pain, with studies reporting rates ranging between 48% and 78% among university students, compared to a general population prevalence rate of 23.1% [3–5].

Among the key risk factors for neck pain in students are sedentary behavior, prolonged screen time, and improper posture, all of which have been exacerbated by the growing dependence on digital devices for daily and academic activities. Studies also suggest that psychological factors such as anxiety and depression influence the occurrence of neck pain [1,3,6].

Prolonged use of mobile phones, laptops, and tablets often results in forward head posture and excessive cervical spine flexion, leading to chronic musculoskeletal discomfort [7]. Furthermore, an observational study has shown that 91% of students practice a flexed neck posture while using mobile devices, emphasizing a strong correlation between screen exposure and neck pain [8]. Besides, a study by Maayah reported that excessive mobile phone usage, combined with improper posture, is a key contributor to chronic neck and shoulder pain [9].

Research suggests that maintaining a neutral spine and minimizing static postures may mitigate the progression of neck pain. However, many students engage in improper study practices, such as sitting slouched forward, reading while in bed, or using chairs without back or neck support, all of which increase the strain on the cervical spine, shoulders, and upper extremities, further progressing musculoskeletal discomfort [10–12].

Despite extensive studies done on digital device usage, limited studies have investigated the specific musculoskeletal impacts of various studying utilities, particularly in relation to posture, duration, and individual differences such as sex, which may play a significant role in neck pain development. With academic institutions including both digital and book based methods, it is important to examine how these study methods affect musculoskeletal health. In addition, after reviewing the literature, we found that there is limited literature that identifies the most ergonomically best utilities and studying positions that minimize neck pain, particularly when comparing various digital devices and books. By investigating the association of both digital and book-based studying with neck pain, it will provide a better understanding of risk factors that contribute to neck pain in students.

This study aims to evaluate the prevalence of neck pain among students at the University of Jordan and to examine the association between individual demographic characteristics, sex, history of neck pain, and postural habits while studying including the use of books and digital devices such as (phones, tablets and laptops) with neck pain severity and duration.

## Methods

### Study area and period

The study was carried out at the University of Jordan between 22 December 2024, and 27 January 2025.

### Study design and population

This study applied a cross-sectional design using a structured questionnaire to assess the prevalence, risk factors, and the severity of neck pain among university students. The study was done through face-to-face interviews including students from all 20 faculties at the University of Jordan regardless of their age, sex, or ethnicity.

### Study population

The study population included all enrolled students enrolled at the University of Jordan during the 2024–2025 academic year. Data were collected through face-to-face interviews with students from all 20 faculties at the University of Jordan, regardless of age, sex, or ethnicity.

### Eligibility criteria

Students were included if they were actively enrolled at the University of Jordan during the study period and agreed to participate. In contrast, students with musculoskeletal diseases, anomalies, or a history of trauma or surgeries in the neck or shoulder regions were excluded from this study.

### Sample size determination

The sample size was calculated using Raosoft online calculator [13]. Assuming a 95% confidence level, 5% margin of error, and 50% estimated prevalence with a total population of around 50,000 students. The minimum sample size required for our study was estimated around 385 students.

### Sampling technique and procedure

A stratified convenience sampling method was utilized to ensure proportional representation across faculties, with each strata comprising a percentage of our sample in correspondence to the number of students in that faculty. The total population consisted of around 50,000 students, from which a final sample size of 507 students was determined. Each faculty represented a stratum, and the number of participants selected from each was proportional to the total number of students enrolled in that faculty. Within each faculty, students were approached in common areas such as lecture halls, corridors, study spaces, cafeterias, and during organized faculty activities. They were invited to participate voluntarily during the data collection period. Participants were selected based on availability rather than through formal probability-based random sampling. Proportional allocation across faculties was used to improve representation of students from different fields of study.

### Data collection tool and procedure

The questionnaire covered demographic factors (age, sex, faculty of study), study-related behaviors such as postural habits while studying, books and digital device usage, study duration, and physical activity. To evaluate neck pain, participants reported details about its onset, pain site and duration throughout the week, and intensity using the Numeric Rating Scale (NRS-11), where students were asked to rate their pain on a scale from 0 to 10, where 0 represents “no pain” and 10 represents “worst pain imaginable” [14].

Additionally, participants were asked about their approaches for managing this pain, including the use of medication and non-medication interventions such as the usage of heat/cold pads, physical therapy or exercise, and behavioral modifications like adjusting posture or reducing screen time. Participants were also asked about their knowledge regarding the relationship between posture and neck pain to evaluate participants’ awareness of how ergonomic factors impact musculoskeletal health. Awareness was assessed using a single direct question: “Are you aware that posture and neck pain are associated?”.

### Operational definitions and measurements

Neck pain severity was measured using a numerical rating scale (0–10), categorized as mild (1–3), moderate (4–6), and severe (7–10). Neck pain duration refers to the period (in days) the participant experienced pain during the previous week.

### Data quality management

The questionnaire was designed primarily to assess the prevalence and risk factors of neck pain related to study posture and digital device use among university students.

The questionnaire validation process included assessments of both face validity and content validity. Four experts from different medical fields including public health, family medicine, anatomy and orthopedics evaluated each item for its domain relevance. The Content Validity Index (CVI) was then calculated. According to [15] when the number of experts is five or fewer, an item-level CVI (I-CVI) of 1.00 is required for adequate content validity [14]. The questionnaire items were modified through expert discussions until they reached complete agreement. The I-CVI was computed by dividing the number of experts who rated an item as relevant by the total number of experts. The scale-level CVI (S-CVI) was obtained as the average of all I-CVI values. [16]

Test–retest reliability was evaluated through 50 participants who took the questionnaire twice during a 9-day period between assessments. The Intraclass Correlation Coefficient (ICC) served as the method to evaluate stability during different time periods. The ICC values followed this interpretation system: values below 0.5 showed poor reliability and values between 0.5 and 0.75 showed moderate reliability and values between 0.75 and 0.9 showed good reliability and values above 0.9 showed excellent reliability [17].

### Data processing and analysis

The statistical software JASP (version 0.19.0.0) was used for data processing and analysis. Descriptive statistics, including frequency and percentage, were calculated for categorical variables, while mean and standard deviation were reported for continuous variables. Univariate and multivariate logistic regression was conducted to estimate the effect of the type of studying utility used on neck pain and pain site and the effect of posture on neck pain utilizing the forced entry selection method. In the model used to estimate the effect of study utilities on neck pain, both shoulders, right shoulder and left shoulder categories were grouped into one category named “shoulder pain”. The selection of the variables in all models was based on prior empirical knowledge and their hypothesized relationship (direction and interactions) between the exposure and outcome, rather than solely on statistical significance. Forward selection was additionally performed during model construction. Specifically, for the model estimating the effect of study devices on neck pain, sex and faculty were included as confounders, and age was included as an independent predictor, while posture and study duration were excluded as they were considered potential mediators. To fully elucidate the relationship, the device model was run three times with different reference categories. A separate model estimating the effect of posture on neck pain included sex, faculty, primary device, and study hours as confounders, with age as an independent predictor. In the third model used to estimate the effect of study device usage and pain site, we included sex as a potential confounder and physical activity and age as independent predictors of the outcome. The results were reported as odds ratio and their 95% confidence interval and a p-value of less than 0.05 was considered statistically significant.

### Ethical approval

The study received ethical approval from the Institutional Review Board (IRB) of the University of Jordan on December 12, 2024 (Decision No. 318/2024), which made certain that every research activity carried out complied with the ethical frameworks and standards. Participants were fully informed about the purpose of this study, and a verbal informed consent was obtained before participation, which was documented through a required confirmation checkbox at the beginning of the online questionnaire. As no identifiable data were collected, responses were confidentially recorded and securely stored to maintain data.

## Results

### Validity and reliability

The questionnaire obtained acceptable face validity. Each item reached an I-CVI score of 1.00 together with an S-CVI of 1.00 which shows complete expert consensus. The test–retest reliability was good based on ICC values above 0.75. The reliability scores for domains 3 and 5 were moderate. S2 Table

### Sociodemographic characteristics of the participants

A total of 556 students were asked to participate in the study, with a 91.2% response rate. Our sample was composed of 266 (52.5%) males and 241 (47.5%) females, aged 18–29 years old, with a mean age of 21 and a standard deviation of 1.93 years, from different faculties across the University of Jordan. Table 1

**Table 1:** Sociodemographic characteristics.

Factor	Total, N = 507*	Males, N = 266*	Females, N = 241*
**Age**	21 ± 1.93	21.27 ± 2.11	20.61 ± 1.64
**Faculty**			
School of Medicine	61 (12%)	26 (42.6%)	35 (57.4%)
School of Engineering	51 (10.1%)	38 (74.5%)	13 (25.5%)
School of Foreign Languages	49 (9.7%)	31 (63.3%)	18 (36.7%)
School of Business	44 (8.7%)	30 (68.2%)	14 (31.8%)
School of Arts	37 (7.3%)	18 (48.6%)	17 (51.4%)
School of Pharmacy	28 (5.5%)	6 (21.4%)	22 (78.6%)
School of Science	27 (5.3%)	15 (55.6%)	12 (44.4%)
King Abdullah II School of Information Technology	27 (5.3%)	21 (77.8%)	6 (22.2%)
School of Agriculture	26 (5.1%)	12 (46.2%)	14 (53.8%)
School of Dentistry	26 (5.1%)	13 (50%)	13 (50%)
School of Shari’a	20 (3.9%)	9 (45%)	11 (55%)
School of Educational Sciences	19 (3.7%)	5 (26.3%)	14 (73.7%)
School of Sport Science	15 (3%)	8 (53.3%)	7 (47.7%)
School of Rehabilitation Sciences	14 (2.8%)	6 (42.9%)	8 (57.1%)
School of Law	14 (2.8%)	4 (28.6%)	10 (71.4%)
School of Nursing	13 (2.6%)	3 (23.1%)	10 (76.9%)
School of Graduate Studies	12 (2.4%)	4 (33.3%)	8 (66.6%)
School of Archaeology and Tourism	10 (2%)	7 (70%)	3 (30%)
School of Arts and Design	9 (1.8%)	5 (55.6%)	4 (44.4%)
Prince Al Hussein Bin Abdullah II School of International Studies	5 (1%)	5 (100%)	0 (0%)

* Mean ± SD, frequency (%)

### Device usage and posture

The majority of students 67.2% spent more than 5 hours a day on their phones for non-studying activities, while 53.6% spent 3 hours or less a day of studying. The most used primary utilities for studying were laptops 31.4% followed by tablets 28.4%, and the least were phones 20.3% and books 19.9%. On the other hand, phones were the most used secondary devices for studying 31.2%. Most of the students were either sitting slouched 37.3% or with partial back support 27.8%, while 16% were sitting with full back support and 3% without any back support. Table 2

**Table 2:** Device usage and posture.

	n (%)
**Hours per day spent on phone**	
Less than 3 hours	49 (9.7%)
3-5 hours	117 (23.1%)
5-7 hours	166 (32.7%)
More than 7 hours	175 (34.5%)
**Study duration**	
Less than 1 hour	89 (17.6%)
1-3 hours	183 (36%)
3-5 hours	142 (28%)
5-7 hours	66 (13%)
More than 7 hours	27 (5.3%)
**Primary studying device**	
Tablet	144 (28.4%)
Phone	103 (20.3%)
Laptop	159 (31.4%)
Books	101 (19.9%)
**Secondary studying device**	
Tablet	29 (5.7%)
Phone	158 (31.2%)
Laptop	87 (17.1%)
Books	74 (14.6%)
None	159 (31.4%)
**Posture when studying**	
Sitting with full back support	81 (16%)
Sitting slouched	189 (37.3%)
Sitting with partial back support	141 (27.8%)
Sitting without back support	15 (3%)
Lying down	62 (12.2%)
Walking	19 (3.7%)

### Prevalence of neck pain

**Fig 1:**
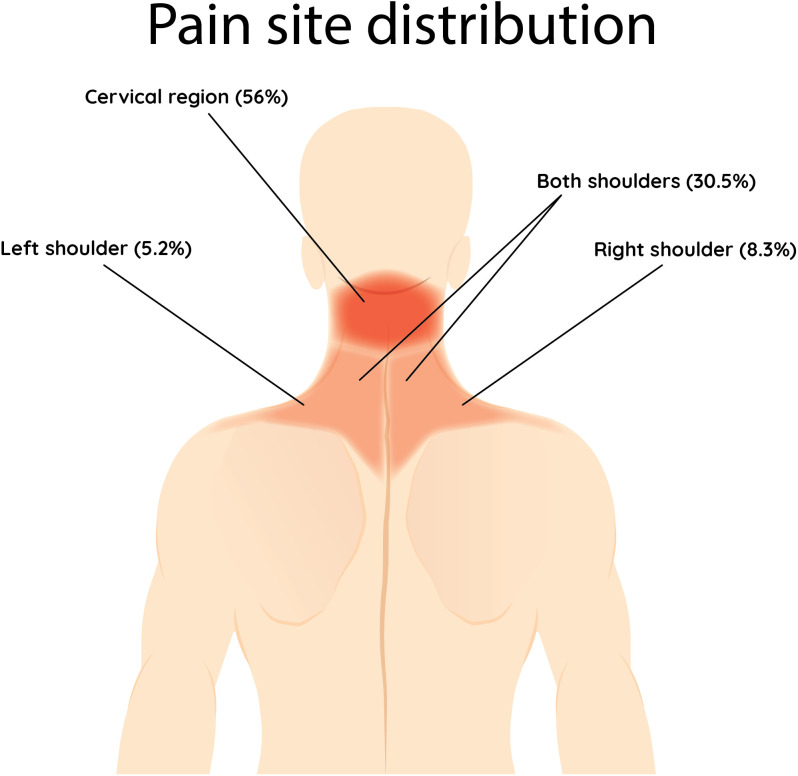
Shows the pain site distribution of the collected sample.

**Fig 2:**
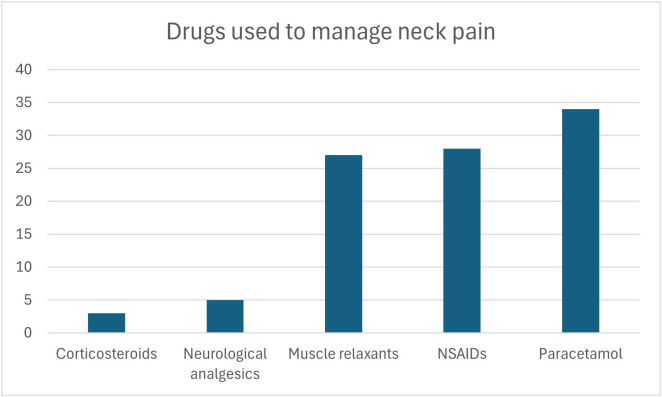
Shows how many students use each medication for neck pain management.

**Fig 3:**
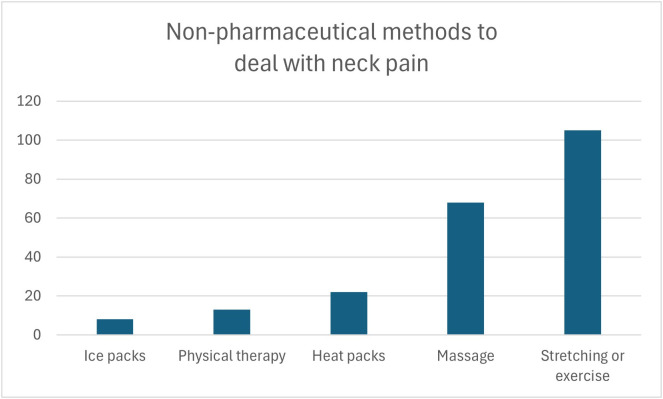
Shows how many students use each non-pharmaceutical method for neck pain management.

Of the 507 students included in our study, 52.4% reported neck pain, with the most common site being the cervical region 56%, followed by both shoulders 30.5%, right shoulder 8.3%, and left shoulder 5.2% Fig 1. 85.6% scored 4 or less on NRS-11, while the rest scored more than 4. Forty and six-tenths percent of the 266 students who reported neck pain stated that their pain affected the duration they spent on studying and 74.4% said that they usually change their posture when experiencing neck pain. 117 (59.1%) of the students who usually change their posture when experiencing neck pain (n = 198) reported improvement of pain, and 71 (35.8%) reported partial improvement. 16.6% of students with neck pain reported using drugs to treat their pain, and 57.5% of students reported usage of non-pharmaceutical methods. The types of both drug used and non-pharmaceutical methods are shown in Fig 2 and Fig 3. Lastly, 86.4% of the included students were aware of the relationship between posture and neck pain. Table 3

**Table 3:** Neck pain prevalence, site, and severity.

	n (%)
**Last time experienced neck pain**	
Today	173 (34.1%)
Last week	93 (18.3%)
Last month	79 (15.58%)
Last 6 months	34 (6.7%)
More than 6 months	49 (9.7%)
I never had neck pain	79 (15.6%)
Pain duration (n = 266)	
Less than 30 min	115 (22.7%)
30 min – 1 hour	39 (7.7%)
1-3 hours	49 (9.7%)
More than 3 hours	63 (12.4%)
**Pain severity**	
≤ 4	434 (85.6%)
> 4	73 (14.4%)
**Effect of pain on studying hours (n = 266)**	
Decline	108 (40.6%)
No effect	158 (59.4%)
**Posture change (n = 266)**	
No	68 (25.6%)
Yes	198 (74.4%)
**Improvement after posture change (n = 198)**	
No	10 (5.1%)
Partially	71 (35.8%)
Yes	117 (59.1%)
Medication usage (n = 266)	
Yes	84 (16.6%)
No	182 (35.9%)
**Complementary therapy usage (n = 266)**	
Yes	153 (57.5%)
No	113 (42.5%)
	
**Physical activity**	
Sedentary	130 (25.6%)
Light activity	195 (38.5%)
Moderate activity	99 (19.5%)
High activity	83 (16.4%)
**Awareness of posture and neck pain relationship**	
Yes	438 (86.4%)
Unsure	50 (9.9%)
No	19 (3.7%)

### Association between neck pain and other risk factors

In females, the occurrence of reporting neck pain was associated with 2.5 times the odds (OR = 2.52, CI [1.76, 3.6], *p* = < 0.001), sitting slouched was associated with double the odds (OR = 2.03, CI [1.2, 3.44], *p* = 0.008), and studying for 5–7 hours a day was associated with threefold higher odds of reporting neck pain(OR = 3.08, CI [1.55, 6.15], *p* = 0.001). On the contrary moderate physical activity was associated with lower odds of reporting neck pain (OR = 0.61, CI [0.37, 0.99], *p* = 0.044). Furthermore, using tablets was associated with higher odds of neck pain reporting by 2.22 times when compared to using phones (OR = 2.22, CI [1.32, 3.71], *p* = 0.002). Interestingly, we found that students at King Abdullah II School of Information Technology faculty had a reduced odd of reporting neck pain when compared to the faculty of medicine. Regarding pain site, in females the odds of reporting pain at the shoulders was 1.87 times higher when compared to the cervical region (OR = 1.87, CI [1.13, 3.09], *p* = 0.014), while using tablets or laptops reduced the odds by 73% (OR = 0.27, CI [0.13, 0.56], *p* = < 0.001) and 68% (OR = 0.32, CI [0.16, 0.66], p = 0.002) when compared to using books and, respectively. The rest of the variables were not statistically associated with either neck pain or pain site Tables 4–6.

**Table 4:** Logistic regression model for the estimate of the effect of device usage on pain site.

	Univariate analysis				Multivariate analysis			
		95% CI				95% CI		
	OR	Lower	Upper	*p*-value	OR	Lower	Upper	*p*-value
**(Intercept)**					0.26	0.01	5.28	0.377
**Age**	1.05	0.93	1.19	0.444	1.08	0.94	1.24	0.267
**Primary device**								
Books	Ref							
Phone	0.6	0.26	1.36	0.22	0.63	0.27	1.5	0.304
Tablet	0.27	0.13	0.56	< 0.001*	0.26	0.12	0.55	< 0.001*
Laptop	0.32	0.16	0.66	0.002*	0.4	0.19	0.85	0.017*
**Sex**								
Male	Ref							
Female	1.87	1.13	3.09	0.014*	1.8	1.02	3.2	0.044*
**Physical activity**								
Sedentary	Ref							
Light activity	1.56	0.87	2.78	0.136	1.27	0.67	2.36	0.445
Moderate activity	0.73	0.34	1.58	0.423	0.73	0.33	1.63	0.446
High activity	0.44	0.18	1.05	0.064	0.44	0.18	1.11	0.083

Books, males, and no physical activity were used as references for primary device usage, sex and physical activity variables, respectively. Shoulder pain was used as the reference for the outcome.

*: Indicates statistical significance

Ref: Reference

**Table 5:** Logistic regression model for the estimate of the effect of device usage on neck pain.

	Univariate analysis				Multivariate analysis			
		95% CI				95% CI		
	OR	Lower	Upper	*p*-value	OR	Lower	Upper	*p*-value
**(Intercept)**					0.19	0.02	2.32	0.193
**Study device**								
Phone	Ref							
Tablet	2.22	1.32	3.71	0.002*	2.68	1.34	5.36	0.005*
Laptop	1.59	0.96	2.62	0.071	2.65	1.45	4.87	0.002*
Books	1.67	0.96	2.91	0.07	1.47	0.79	2.75	0.224
**Study device**								
Books	Ref							
Tablet	1.33	0.79	2.22	0.279	1.82	0.91	3.65	0.092
Laptop	0.95	0.58	1.57	0.842	1.8	0.97	3.37	0.064
Phone	0.6	0.34	1.04	0.07	0.68	0.36	1.27	0.224
**Study device**								
Tablet	Ref							
Phone	0.45	0.27	0.76	0.002*	0.37	0.19	0.75	0.005*
Laptop	0.72	0.45	1.13	0.151	0.99	0.54	1.82	0.977
Books	0.75	0.45	1.26	0.279	0.55	0.27	1.1	0.092
**Age**	1.02	0.93	1.12	0.642	1.06	0.94	1.19	0.328
**Sex**								
Male	Ref							
Female	2.52	1.76	3.6	< 0.001*	2.44	1.62	3.7	< 0.001*
**Faculty**								
School of medicine	Ref							
School of Business	2.07	0.92	4.66	0.077	4.05	1.58	10.36	0.004*
School of Law	3.55	0.9	13.99	0.07	6.09	1.34	27.73	0.019*
School of Arts and Design	3.39	0.65	17.63	0.147	6.16	1.04	36.56	0.046*
**Physical activity**								
Sedentary	Ref							
Light activity	1.36	0.86	2.14	0.184	–	–	–	–
Moderate activity	0.61	0.37	0.99	0.044*	–	–	–	–
High activity	0.63	0.38	1.06	0.08	–	–	–	–

Tablets, males, and the school of medicine were used as references for primary device usage, sex, and faculty variables, respectively. The absence of neck pain was used as the reference for the outcome. Only the faculties that were associated with neck pain are presented in the table.

*: Indicates statistical significance

Ref: Reference

**Table 6:** Logistic regression model for the estimate of the effect of posture on neck pain.

	Univariate analysis				Multivariate analysis			
		95% CI				95% CI		
	OR	Lower	Upper	*p*-value	OR	Lower	Upper	*p*-value
**(Intercept)**					0.08	0.01	1.24	0.071
**Posture**								
Sitting upright with full back support	Ref							
Sitting slouched	2.03	1.2	3.44	0.008*	2.18	1.18	4.04	0.013*
Sitting upright with partial back support	0.9	0.52	1.56	0.706	1.25	0.66	2.37	0.499
Sitting without back support	0.83	0.27	2.56	0.75	1	0.28	3.62	0.996
Lying down	1.62	0.83	3.16	0.156	2	0.92	4.34	0.079
Walking	2.71	0.94	7.83	0.066	2.33	0.7	7.71	0.166
**Sex**								
Male	Ref							
Female	2.52	1.76	3.6	< 0.001*	2.18	1.4	3.37	< 0.001*
**Faculty**								
School of medicine	Ref							
School of Business	2.07	0.92	4.66	0.077	4.76	1.74	13	0.002*
School of Arts	1.27	0.56	2.89	0.568	3.01	1.03	8.8	0.044*
King Abdullah II School of Information Technology	0.28	0.1	0.78	0.015*	0.61	0.18	2.06	0.43
School of Law	3.55	0.9	13.99	0.07	6.89	1.43	33.13	0.016*
School of Arts and Design	3.39	0.65	17.63	0.147	8.95	1.45	56	0.018*
Study device								
Tablets	Ref							
Books	0.75	0.45	1.26	0.279	0.58	0.28	1.19	0.134
Laptops	0.72	0.45	1.13	0.151	1.04	0.56	1..95	0.905
Phone	0.45	0.27	0.76	0.002*	0.39	0.19	0.81	0.011*
**Study duration**								
less than 1 hour	Ref							
1-3 hours	0.83	0.5	1.38	0.475	0.71	0.39	1.28	0.25
3-5 hours	1.34	0.79	2.28	0.28	1.02	0.52	1.98	0.96
5-7 hours	3.08	1.55	6.15	0.001*	2.31	0.98	5.44	0.056
More than 7 hours	1.34	0.56	3.18	0.51	0.83	0.29	2.38	0.728
**Age**	1.02	0.93	1.12	0.642	1.07	0.95	1.21	0.258

Sitting upright with full back support, males, school of medicine, tablets, and less than 1 hour were used as references for primary device usage, sex, faculty, primary study device, and study duration variables, respectively. “No neck pain” was used as the reference for the outcome. Only the faculties that were associated with neck pain are presented in the table.

*: Indicates statistical significance

### Multivariate analysis

To further investigate the effect of primary device usage on neck pain and pain site and posture on neck pain, we built a logistic regression model.

We found that in comparison to using books as a study device, using tablets was associated with lower odds of experiencing cervical pain compared to shoulder pain by approximately 74% (OR = 0.261, CI [0.12, 0.55], p < 0.001). Moreover, using a laptop as a study device was associated with lower odds of experiencing cervical pain compared to shoulder pain by 60% (OR = 0.4, CI [0.19, 0.85], p = 0.017). Table 4

Using laptops (OR = 2.65, CI [1.45, 4.87], p = 0.002) or tablets (OR = 2.68, CI [1.34, 5.36], p = 0.005) for studying was associated with higher odds of reporting neck pain nearly 2.7 times when compared to using phones. Meanwhile, there wasn’t any statistically significant evidence that studying using books was associated with higher or lower odds of reporting pain when compared with the rest of devices, (tablets (OR = 1.82, CI [0.91, 3.65], p = 0.092), laptops (OR = 1.8, CI [0.97, 3.37], p = 0.064) and phones (OR = 0.68, CI [0.36, 1.27], p = 0.224)), respectively. Moreover, we did not find a statistically significant change in the odds between tablet users and laptop users. Table 5

Sitting slouched was associated with higher odds of reporting neck pain twice compared to sitting upright with full back support (OR =2.18, CI [1.18, 4.04], p = 0.013). Table 6

## Discussion

In this study, our sample revealed that most of the students at the University of Jordan have experienced neck pain during the week prior to the interview, with several factors showing a statistically significant association. Multivariate analysis showed that students who studied for 5–7 hours per day had an odds ratio of OR = 2.31, CI [0.98, 5.44] for reporting neck pain compared to those who studied for shorter durations. Similarly, Zheng [18] an observational cross-sectional study among university students found that those using electronic devices for more than six hours daily had 2.01 times higher odds [95% CI: 1.11–3.65] of reporting neck pain than those with less exposure.

Female students were 2.18 CI [1.4, 3.37] times as likely to report neck pain as male students. Furthermore, multivariate analysis showed students who studied on tablets were associated with higher odds of reporting neck pain by 2.68 times compared to those who studied on mobile phones.

Apart from faculty and lack of physical activity being linked with neck pain, sitting in a slouched posture was associated with twofold higher odds of reporting neck pain.

All of the aforementioned factors were significantly associated with neck pain in this sample. Our findings reveal a growing incidence of neck pain among students, particularly following the widespread shift to digital-based learning and remote work during the COVID-19 pandemic. Previously, the prevalence among university students was 37.3%, rising to 62.7% after the transition [18]. This aligns with prior research linking prolonged digital device use to musculoskeletal issues. In our study of 507 students, 52.4% reported neck pain in the past week, primarily in the cervical region, followed by the shoulders. Laptop and tablet users showed higher pain prevalence than mobile users, and consistent with earlier studies, females reported higher pain levels than males.

Our findings of neck pain prevalence among university students align with studies that evaluated neck pain in 7 days. For example, a study done among medical students at the University of Ankara, Turkey, reported a 50.6% prevalence of neck pain [19], which closely matches our observed result of 52.4%. Similarly, a Canadian study also found comparable one-week prevalence rates, ranging from 63.7% among faculty of Health sciences students, 45.4% among Education faculty students, to 76.9% among chiropractic students [20].These variations could be attributed to differences in device usage habits, culture, ergonomic awareness, and educational interventions.

The increasing reliance on digital devices has significantly influenced both work and study environments, requiring users to engage in suboptimal postures during device usage, which may contribute to a higher likelihood of neck pain. [5,18]. Supporting this observation, a meta analysis by Gao reported that university students who used digital devices for extended periods had 53% higher odds of developing neck pain [4].

Our multivariate analysis revealed that tablet and laptop users had nearly 2.7 times greater odds of reporting neck pain when compared to phone usage. This finding suggests that studying on mobile phones was associated with lower reported odds of neck pain compared with tablets and laptops, while no meaningful difference was observed compared with studying from books.

In regards to the site of neck pain, tablet use was also strongly associated with shoulder pain reporting, with nearly four times higher odds of reporting shoulder pain than neck pain when compared to using books.

These results indicate that students who primarily used mobile phones reported lower prevalence of musculoskeletal discomfort compared with tablet and laptop users in this sample. Despite their small screen size and suboptimal ergonomic design, mobile phones turned out to be a better study utility compared to laptops or tablets. One study found that phone users tend to adjust their postures more frequently than laptop and tablet users, reducing static muscle load and strain. Therefore, reducing the risk of neck pain development [21].

Postural habits were another significant risk factor for neck pain in our study. Our results showed that students who maintained a slouched posture had 118% higher odds of reporting neck pain compared with those who sat upright with full back support. This result aligns with multiple studies that have associated forward bent posture and prolonged static postures with higher odds of neck pain occurrence. A study by Cagnie reported that frequently having the neck in a forward bent posture for prolonged durations was associated with higher odds of developing neck pain by two times, with prolonged sitting further associated with nearly twofold higher odds as well [21]. Similarly, a meta-analysis by Gao further supports these findings, reporting an odds ratio of 2.04 for prolonged head-bowing, which contributes to neck pain development [4]. Overall, these studies emphasize that practicing improper postures, particularly slouched posture, is associated with higher cervical spine stress and linked to a higher occurrence of neck pain. In our sample, female students were associated with a higher likelihood of reporting neck pain than males [22], consistent with previous research highlighting sex differences in musculoskeletal complaints. For instance, a study in a Chinese high school showed that neck/shoulder pain and low back pain were reported more frequently by females than males [23,24], and Salameh [22] found that among Jordanian medical students, females were associated with higher odds of text neck syndrome than males. These differences may be attributed to biological factors, including higher nociceptor density in women’s skin and muscles, which is linked to higher pain sensitivity, and hormonal fluctuations, where high estrogen levels are associated with a pro-nociceptive effect while testosterone exhibits an association with an anti-nociceptive, protective effect [25]. Sociocultural factors also contribute, as expressing pain is more socially acceptable among women, leading to higher self-reported rates of neck pain compared to men [26].

An interesting finding was that the majority of students (67.2%) spent more than 5 hours per day on their phones without studying, while (53.6%) of students spent 3 hours or less daily on studying. These findings show that many students spend a large amount of time on distractions rather than studying, which may reduce their productivity, competency, and academic responsibility. Although this study did not assess academic performance or mental health outcomes, prior research has reported associations between prolonged smartphone use and poor sleep, anxiety, stress, and lower academic performance [27, 28].

In this study, we investigated the prevalence of neck pain and how specific postures and the use of certain studying utilities contribute to the risk of reporting this condition. Contrary to several studies conducted in the region, our study employed multivariate methods for data analysis, which reduced the degree of bias introduced by confounders. Furthermore, in contrast to a study [27,29] which was conducted at the ‌‌University of Jordan, specifically the health care faculties, the data utilized in our study was collected through face-to-face interviews, allowing participants to seek clarification when needed and helping to minimize incomplete or inconsistent responses. Moreover, we stratified our sample population across all faculties to increase the generalizability of the‌‌ data.

## Limitations

The study included only University of Jordan students, limiting the generalizability to other people in Jordan. Additionally, we acknowledge that our reliance on self-repo‌‌rted data for both study posture and neck pain symptoms might introduce recall bias. It is possible that students who were already suffering from pain at the time of the interview tended to over report their posture compared to those without pain. While we believe this does not change the overall direction of our findings, it suggests that the associations we found should be interpreted as subjective perceptions rather than absolute clinical measures. Future research using direct observational methods could help validate these student reported postures.

The cross-sectional design could not determine the direction of causality between these variables. Although we stratified our sample, we used a stratified convenience sampling approach in which participation was voluntary and dependent on students’ availability during data collection, which may have caused selection bias. Thus, the prevalence estimates and associations that were observed may not be fully generalizable to all university students.

## Conclusion

We found that most students had neck pain in the week before the interview. Sitting slouched while studying with common study devices was associated with more than two times the odds of reporting neck pain compared to sitting upright with full back support. Tablet devices were associated with approximately three times higher odds of reporting neck pain than phones. There was no statistical difference between using books, tablets, and laptops. Additionally, using tablets and laptops as studying utilities was associated with higher odds of reporting pain in the shoulder region more than the cervical region when compared to studying from books.

### Recommendations

Based on the study findings, sitting positions and studying devices were associated positively with neck pain which is a rising problem among students, several practical recommendations are suggested to reduce the prevalence of neck pain in society: Universities and educational institutions should implement educational workshops and awareness activities on proper sitting position during studying, check and improve study areas, library and classroom chairs to provide neck support. Education authorities and local health should collaborate with universities to develop programs that prevent neck pain by setting basic rules and guidelines for safe study environments. Further researchers should conduct long term studies to examine how sitting positions and studying devices affect neck pain over time, test the effectiveness of the workshops, programs and awareness activities in reducing neck pain.

## Supporting information

S1 File Neck Pain Questionnaire. (DOCX)

S1 Table Neck Pain Data. (XLSX)

S2 Table ICC values for the test-retest reliability of the questionnaire. (DOCX)
