# Supplementary material for: The prevalence of neck pain and its association with studying device usage and posture among students at the University of Jordan: A cross-sectional study
Source: PLoS One. 2026 May 22;21(5):e0326478. doi: 10.1371/journal.pone.0326478 (PMC13196932; doi:10.1371/journal.pone.0326478)
Supplement: S1 File — (DOCX) [file pone.0326478.s001.docx]

Neck pain questionnaire

Domain 1: Device usage patterns

Q1. How many hours per day do you typically spend using your mobile phone?

- Less than 1 hour
- 1–3 hours
- 3–5 hours
- 5–7 hours
- More than 7 hours

Q2. What is your primary device used for studying or academic activities?

- Smartphone
- Laptop
- Tablet
- Desktop computer
- Printed books or notes
- Other: __________

Q3. What is your secondary device used for studying (if any)?

- Smartphone
- Laptop
- Tablet
- Desktop computer
- Printed books or notes
- None
- Other: __________

Q4. Which posture best describes your typical position while using your studying device?

- Sitting upright with full back and head support
- Sitting upright with partial back support (no head support)
- Sitting without back support (e.g., stool or edge of bed)
- Sitting slouched or leaning forward
- Standing
- Walking
- Lying down
- Other: __________

Q5. On average, how many hours per day do you spend using your studying device?

- Less than 1 hour
- 1–3 hours
- 3–5 hours
- 5–7 hours
- More than 7 hours

Domain 2: Pain Characteristics

Q6. When did you last experience neck pain?

- Today
- Within the past week
- Within the past month
- Within the past 6 months
- More than 6 months ago
- I have never experienced neck pain

Q7. Over the past week, how often did you experience neck pain?
(0 = No pain at all, 10 = Pain occurs very frequently)

- 0 1 2 3 4 5 6 7 8 9 10

Q8. Where do you usually feel the pain?

- Cervical region
- Right shoulder
- Left shoulder
- Both shoulders
- I do not experience pain

Q9. How long does your neck pain usually last each time it occurs?

- Less than 30 minutes
- 30 minutes – 1 hour
- 1–3 hours
- More than 3 hours
- I do not experience neck pain

Q10. On a scale from 0 to 10, how would you rate your current neck pain intensity?
(0 = No pain, 10 = Worst pain imaginable)

- 0 1 2 3 4 5 6 7 8 9 10

Domain 3: Pain Management

Q11. Do you currently take any medication for neck pain?

- Yes
- No

Q12. If yes, which type(s) of medication do you use? *(Select all that apply)*

- Nonsteroidal Anti-Inflammatory Drugs (e.g., Ibuprofen, Naproxen, Aspirin)
- Paracetamol (e.g., Panadol, Tylenol)
- Corticosteroids
- Neuropathic pain medication (e.g., Gabapentin, Amitriptyline)
- Opioids
- Muscle relaxants (e.g., Tizanidine, Baclofen, Orphenadrine)
- I do not use any medication
- Other: __________

Q13. How frequently do you use these medications?

- Rarely (less than once per week)
- Occasionally (1–2 days per week)
- Frequently (3–5 days per week)
- Daily (6–7 days per week)

Q14. Which non-medication treatments have you used to relieve neck pain? *(Select all that apply)*

- Heat therapy (e.g., warm compress, heating pad)
- Cold therapy (e.g., ice packs)
- Massage
- Stretching or neck exercises
- Physical therapy sessions
- None
- Other: __________

Domain 4: Behavioral and Postural Adjustments

Q15. After developing neck pain, did you reduce your screen or device usage?

- Yes
- No
- I do not experience neck pain

Q16. After developing neck pain, did you modify your posture or sitting position while using your device?

- Yes
- No
- I do not experience neck pain

Q17. Did these changes (if any) help improve your neck pain?

- Yes
- No
- Partially
- I do not adjust my posture or sitting position when experiencing neck pain
- I do not experience neck pain

Domain 5: Awareness of the relationship between posture and neck pain

Q18. Are you aware that prolonged device use or poor posture can contribute to neck pain?

- Yes
- No
- Not sure

Domain 6: Physical activity level

Q19. Which of the following best describes your overall level of physical activity?

- Sedentary (little or no regular exercise)
- Light activity (occasional walking or light sports)
- Moderate activity (exercise or sports 1–3 times per week)
- High activity (exercise or sports more than 3 times per week
