# Supplementary material for: The prevalence of neck pain and its association with studying device usage and posture among students at the University of Jordan: A cross-sectional study
Source: PLoS One. 2026 May 22;21(5):e0326478. doi: 10.1371/journal.pone.0326478 (PMC13196932; doi:10.1371/journal.pone.0326478)
Supplement: S2 Table — (DOCX) [file pone.0326478.s003.docx]

Table 2 ICC values for the test-retest reliability of the questionnaire

|  |  |  |  | *95% CI* | |
| --- | --- | --- | --- | --- | --- |
| Type | Mean ± SD (1) | Mean ± SD (2) | Point Estimate | Lower | Upper |
| Domain 1 | 9.82 ± 2.66 | 9.64 ± 2.41 | 0.859 | 0.765 | 0.917 |
| Domain 2 | 10 ± 3.57 | 10.68 ± 3.54 | 0.69 | 0.512 | 0.811 |
| Domain 3 | 3.72 ± 4.61 | 3.72 ± 4.38 | 0.89 | 0.815 | 0.936 |
| Domain 4 | 4.64 ± 2.21 | 4.72 ± 2.4 | 0.865 | 0.774 | 0.921 |
| Domain 5 | 0.96 ± 0.2 | 0.98 ± 0.14 | 0.662 | 0.474 | 0.793 |
| Domain 6 | 1.34 ± 0.94 | 1.42 ± 0.91 | 0.93 | 0.879 | 0.96 |
| Total score | 30.48 ± 5.92 | 31.16 ± 5.85 | 0.77 | 0.629 | 0.862 |
|  | | | | | |
